# Circulating nesfatin-1 levels in women with polycystic ovary syndrome: A systematic review and meta-analysis

**DOI:** 10.18502/ijrm.v21i10.14533

**Published:** 2023-11-24

**Authors:** Seyed Sobhan Bahreiny, Akram Ahangarpour, Ali Asghar Hemmati, Razieh Kazemzadeh, Mohammad-Navid Bastani, Mohammad Reza Dabbagh, Mojtaba Aghaei

**Affiliations:** ^1^Student Research Committee, Ahvaz Jundishapur University of Medical Sciences, Ahvaz, Iran.; ^2^Medical Basic Sciences Research Institute, Physiology Research Center, Department of Physiology, School of Medicine, Ahvaz Jundishapur University of Medical Sciences, Ahvaz, Iran.; ^3^Marine Pharmaceutical Research Center, School of Pharmacy, Ahvaz Jundishapur University of Medical Sciences, Ahvaz, Iran.; ^4^Department of Biology, Faculty of Science, Shahid Chamran University of Ahvaz, Ahvaz, Iran.

**Keywords:** Polycystic ovary syndrome, Insulin resistance, Body mass index, Meta-analysis.

## Abstract

**Background:**

Polycystic ovary syndrome (PCOS) is a prevalent endocrine disorder in females. Nesfatin-1 is a neuropeptide synthesized in the hypothalamus and other peripheral organs, and there are conflicting opinions about its correlation with PCOS.

**Objective:**

This study aims to investigate the correlation between nesfatin-1 and PCOS and evaluates the effectiveness of nesfatin-1 as a biomarker for the detection of PCOS in women.

**Materials and Methods:**

A systematic review and meta-analysis were conducted to identify pertinent articles from databases such as PubMed, Web of Science, Cochrane, EMBASE, Scopus, and Google Scholar. The standardized mean difference (SMD) and 95% confidence interval (CI) were calculated using a random effects model to compare group outcomes. Additionally, meta-regression and subgroup analysis were performed to elucidate sources of heterogeneity.

**Results:**

The meta-analysis involved 12 studies with 1222 participants, and the findings revealed a significant relationship between PCOS and nesfatin-1 levels. The pooled (SMD = 0.54; 95% CI: 0.00-1.07; p = 0.04) indicated a significant difference between the evaluated groups. Moreover, a subgroup analysis showed that there was a substantial difference in nesfatin-1 levels among women with PCOS and higher homeostatic model assessment for insulin resistance ratio (SMD = 1.46; 95% CI: 0.92-2.00; p < 0.001).

**Conclusion:**

Our meta-analysis indicates an association between high nesfatin-1 levels and PCOS. This suggests a potential role of nesfatin-1 in the development of PCOS and proposes it as a potential diagnostic biomarker for the disease. However, further research is necessary to validate these findings.

## 1. Introduction

Polycystic ovary syndrome (PCOS) is a frequently encountered endocrine disorder affecting women during their reproductive years (1). The prevalence of PCOS alters among different races and countries depending on environmental and genetic factors. This syndrome is distinguished by irregular ovulation, hyperandrogenemia, and reduced fertility. It is often associated with metabolic conditions such as hyperinsulinism, obesity, and insulin resistance (IR) (2). Obesity is a critical concern in women diagnosed with PCOS, as it constitutes the most common clinical problem affecting a significant proportion of patients (3, 4). Furthermore, IR is an important index of interest in research studies concerning women with PCOS.

Research indicates that high insulin levels are directly associated with the severity of the disease (5, 6). However, the pathophysiology of PCOS remains complex and unclear, regulated by various hormones, and possibly influenced by genetic and environmental factors (7, 8). Peptides responsible for the feeling of satiety in the hypothalamus have demonstrated their effectiveness in regulating energy balance, glucose metabolism, and obesity.

In addition, they have a significant impact on gonadal function, playing a crucial regulatory role in both physiological and pathological processes in the ovaries (9). Nesfatin-1 is an example of an anorexigenic peptide that could be linked to the pathophysiology of PCOS (10, 11). Nesfatin-1 derives from the nucleobindin-2 gene and contains 82 amino acids.

The primary source of this hormone is the central nervous system, especially the hypothalamus. Nesfatin-1 is also expressed in peripheral systems such as adipocytes, gastro-endocrine cells, and pancreatic beta cells (12, 13). It has been identified as having a role in regulating feeding behavior and energy metabolism (14). Furthermore, it has been observed to have an anorexic effect by decreasing the frequency of meals and increasing the time between meals (15). Numerous studies have revealed that nesfatin-1 is closely linked to body mass index (BMI), glucose, insulin metabolism, and IR (16, 17). The significant correlation between obesity and IR with PCOS has prompted extensive research into the involvement of nesfatin-1 in energy metabolism, insulin secretion, and food intake (18, 19).

Interestingly, different studies have reported varied findings regarding the levels of nesfatin-1 in women with PCOS, while some studies indicate higher levels (20, 21); others suggest lower levels (22).

This study aims to perform a systematic review and meta-analysis to analyze the variations in nesfatin-1 levels between individuals diagnosed with PCOS and healthy controls. The main objective is to accurately assess the association between nesfatin-1 and PCOS disease.

## 2. Materials and Methods

### Literature search 

In this systematic review and meta-analysis, we conducted a literature search to identify articles related to the research topic. This review was conducted based on PRISMA statement (23). We searched the PubMed, Web of Science, Scopus, Cochrane, EMBASE, and Google Scholar databases for articles published in English from January 2000-April 2023. The search terms included PCOS (polycystic ovary syndrome, PCOS, PCO) and “Nesfatin-1” or “Nesfatin-1 protein” or “NEFA protein” or “NUCB2 protein” or “nucleobindin 2 protein”, and the search was limited to humans. We preselected all articles that were potentially related to PCOS.

### Study selection

The relevant observational studies were selected and reviewed manually and independently by 2 principal investigators, without relying on any automation tools. In addition, the researchers thoroughly reviewed the reference lists of identified articles to ensure that all relevant studies were included. In instances where discrepancies arose, they were resolved through discussions or consultations with a third supervising researcher to ensure the accuracy and quality of the study selection process.

The inclusion criteria for the studies analyzed in this research were as follows: “(i) publication in the English language, (ii) diagnosis of PCOS in women of reproductive age according to the Rotterdam criteria, and (iii) measurement of nesfatin-1 as one of the biomarkers”.

The following exclusion criteria were applied: (i) studies with inaccessible full texts, (ii) studies that did not report the level of nesfatin-1, (iii) studies with insufficient data, (iv) conference proceedings, reviews, randomized controlled trials, and animal studies, (v) studies without control groups.

### Data extraction and quality assessment

The following data were extracted according to a pre-prepared checklist: origin of study characteristics (year of publication, name of first author, and location of study), study design (cross-sectional, cohort, or case-control), participant characteristics (age, number of participants, and BMI), measurements, diagnostic criteria, and reported outcomes. These details are shown in table I.

The quality of the final studies was assessed using the Newcastle-Ottawa scale, which is commonly used to assess nonrandomized studies in meta-analyses. The article consists of 8 sections categorized into 3 main areas: selection (comprising 4 points), comparison (comprising 2 points), and exposure (comprising 3 points) (24). The scoring scale ranged from 0-9, where studies scoring ≤ 4 were categorized as low quality, while those scoring ≥ 5 were classified as high quality. These classifications are presented in table II.

**Table 1:** Characteristics of the studies included in the systematic review and meta-analysis


**Author, year (Ref)**	**Country**	**Number of participants**	**Age (yr)** **(PCOS vs. controls)**	**BMI (kg/m^2^)** **(PCOS vs. controls)**	**Sample (unit)**
**Hamed ** ***et al.*** **, 2022** **(25)** *	Egypt	PCOS: 60 Control: 24	28.42 ± 4.34 30.13 ± 3.26	31.32 ± 4.80 25.43 ± 1.44	Serum (pg/ml)
**Faeza ** ***et al.*** **, 2023** **(26)** *	India	PCOS: 40 Control: 40	26.7 ± 2.6 27.1 ± 3.1	25.07 ± 0.56 27.1 ± 0.62	Serum (ng/ml)
**Wang ** ***et al.*** **, 2021 (27)** *	China	PCOS: 200 Control: 150	28.5 ± 10.1 29 ± 12.1	N/A	Serum (mg/ml)
**Varli ** ***et al.*** **, 2021 (22)** *	Turkey	PCOS: 41 Control: 40	27.7 ± 3.6 29.0 ± 3.7	24.8 ± 4.2 23.7 ± 5.0	Serum (mmol/L)
**Ali ** ***et al.*** **, 2021 (28)** **	Iraq	PCOS: 45 Control: 40	29.3 ± 5.7 29.5 ± 5.2	30.1 ± 4.2 29.3 ± 5.1	Serum (ng/ml)
**Demir ** ***et al.*** **, 2021** **(29)** *	Turkey	PCOS: 44 Control: 40	26.41 ± 5.036 28.23 ± 5.09	24.07 ± 2.97 24.7 ± 3.7	Serum (ng/ml)
**Taskin ** ***et al.*** **, 2016** **(18)** *	Turkey	PCOS: 32 Control: 26	24.72 ± 4.30 26.85 ± 5.06	23.83 ± 3.55 22.16 ± 2.47	Serum (mmol/L)
**Sahin ** ***et al.*** **, 2015 (21)** *	Turkey	PCOS: 54 Control: 48	22.2 ± 4.2 21.5 ± 4.5	30.0 ± 7.5 29.7 ± 5.6	Serum (ng/ml)
**Alp ** ***et al.*** **, 2015 (15)** *	Turkey	PCOS: 55 Control: 35	25.95 ± 5.61 28.14 ± 6.76	24.03 ± 5.06 22.34 ± 3.22	Serum (ng/ml)
**Binnetoglu ** ***et al.*** **, 2014** **(30)** *	Turkey	PCOS: 37 Control: 28	25 ± 7.8 28 ± 6.17	25.17 ± 4.9 22.81 ± 3.6	Plasma, Serum (pg/ml)
**Ademoglu ** ***et al.*** **, 2014** **(11)** **	Turkey	PCOS: 55 Control: 28	25.1 ± 5.6 26.2 ± 4.9	27.4 ± 6.8 21.0 ± 2.8	Plasma, Serum (pg/ml)
**Deniz ** ***et al.*** **, 2012 (9)** *	Turkey	PCOS: 30 Control: 30	23.56 ± 4.80 23.16 ± 3.66	25.03 ± 0.86 24.43 ± 0.50	Serum (ng/ml)
*Case-control study, **Cross-sectional study. BMI: Body mass index, PCOS: Polycystic ovary syndrome					

**Table 2:** Quality assessment based on the Newcastle-Ottawa scale of studies included in this meta-analysis


	**Selection**				**Comparability**	**Exposure**			
**Author, year (Ref)**	**An adequate definition of case**	**Representativeness of the case**	**Selection of controls**	**Definition of controls**	**Control for an important factor**	**Assessment of exposure**	**The same method of ascertainment for cases and controls**	**Non-response rate**	**Score**
	**Hamed ** ***et al.*** **, 2022 (25)**	★	★	★	★	★★	–	★		–	7
**Faeza ** ***et al.*** **, 2023 (26)**	★	–	★	★	★	–	★	–	5
**Wang ** ***et al.*** **, 2021 (27)**	★	★	★	★	★★	–	★	–	7
**Varli** *** et al.*** **, 2021 (22)**	★	★	★	★	★	–	★	–	6
**Ali ** ***et al.*** **, 2021 (28)**	★	★	★	★	★	★	–	6
**Demir ** ***et al.*** **, 2021 (29)**	★	–	★	★	★	–	★	–	5
**Taskin ** ***et al.*** **, 2016 (18)**	★	★	★	★	★★	–	★	–	7
**Sahin ** ***et al.*** **, 2015 (21)**	★	★	★	★	★	–	★	–	6
**Alp ** ***et al.*** **, 2015 (15)**	★	★	★	★	★	–	★★	–	7
**Binnetoglu ** ***et al.*** **, 2014 (30)**	★	★	★	★	★	–	★	–	7
**Ademoglu ** ***et al.*** **, 2014 (11)**	★	- ★	★	★★	–	★	–	6
**Deniz ** ***et al.*** **, 2012 (9)**	★	- ★	★	★	–	★	–	5

### Statistical analysis

The relationship between serum nesfatin-1 level and women with PCOS was assessed using standardized mean difference (SMD) with 95% confidence intervals (CI). The statistical significance of heterogeneity was defined by considering p < 0.05 or I^2^ > 50%, and a random-effects model was utilized. Cochran's Q test and I^2^ statistics were used to assess heterogeneity between studies, and sources of heterogeneity were identified by subgroup analysis and meta-regression. Sensitivity analysis was conducted by individually removing each study to evaluate their impact on the overall results. Statistical analysis was performed using comprehensive meta-analysis version 3 software, developed by Biostat Inc., USA, in 2018. The presence of publication bias was assessed using the Begg's and Egger's tests. The significance level was set at p < 0.05. A prediction interval (PI) was also calculated to estimate the degree of heterogeneity. This interval determines the expected range in which approximately 95% of the true effects should be found.

## 3. Results

### Features of the included studies 

**Figure 1:**
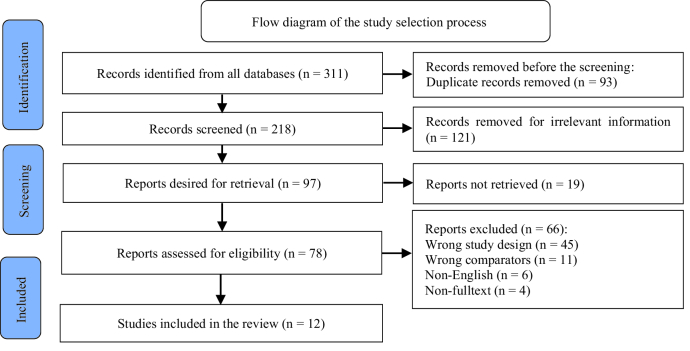
Flow diagram of study selection adjusted by PRISMA.

The search strategy employed retrieved 311 articles from the database; 93 duplicates were eliminated before the screening stage. Further screening of titles and abstracts led to the exclusion of 121 articles. The remaining 97 potentially relevant articles underwent a thorough full-text review. Of these, 56 studies were excluded because of a discrepancy between their study design and the comparison group, 10 records were excluded due to discrepancies in input languages other than English, and other records were omitted because crucial information was not available. Therefore, only 12 studies with 1222 participants met our selection criteria, as shown in figure 1.

### Relationship between serum nesfatin levels and PCOS

#### Meta-analysis

A total of 12 studies were included in the analysis, which reported changes in nesfatin-1 levels in both PCOS groups and corresponding control groups.

**Figure 2:**
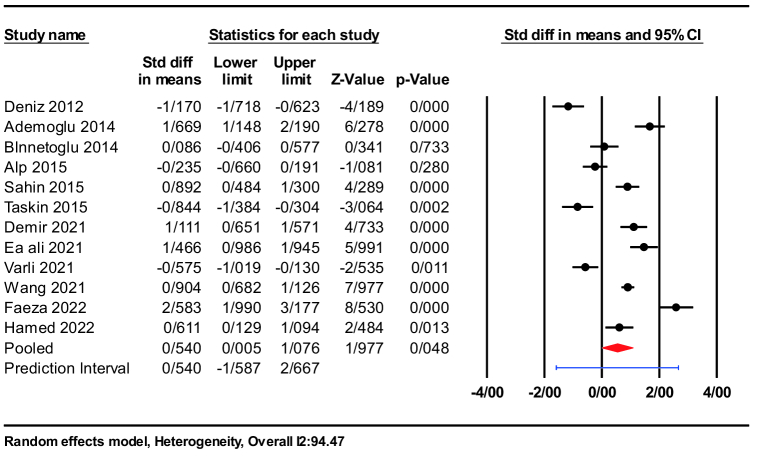
The forest plots compare nesfatin-1 levels between PCOS and control groups.

The strength of the association was measured by calculating the SMD. The result showed a higher nesfatin-1 level in the PCOS group compared with the control group (SMD = 0.54; 95% CI: 0.00-1.07; p = 0.04). In addition, a significant heterogeneity was observed between studies (I ${}^{2}$ = 94.47%; p < 0.001) (Figure 2). These results show that women with PCOS can have high and variable levels of nesfatin, but the results should be interpreted cautiously depending on different conditions.

#### PI

**Figure 3:**
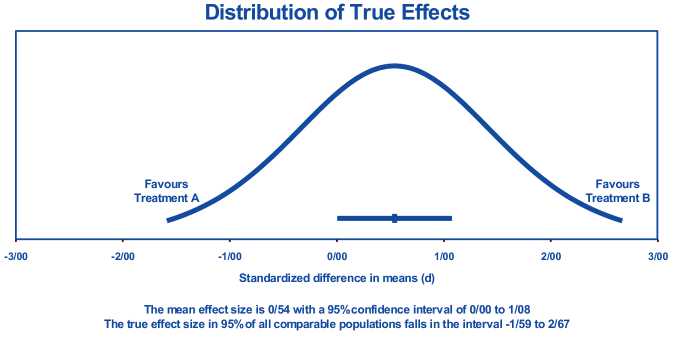
The prediction interval SMD of nesfatin-1 levels between PCOS and control groups.

If we assume that the actual effects conform to normal distribution (in d units), our estimation suggests that the PI ranges from -1.59-2.67. This interval implies that the true effect size in 95% of all comparable populations would lie within this range. In other words, there is a high possibility of the actual effect size in each population falling within this interval. It is important to note that this PI provides a range of plausible values for the true effect size rather than a point estimate. This means the true effect size may vary within this interval based on factors such as sample size, study design, and population characteristics. Hence, caution must be exercised while interpreting the findings within this PI (Figure 3).

#### Subgroup analysis

**Figure 4:**
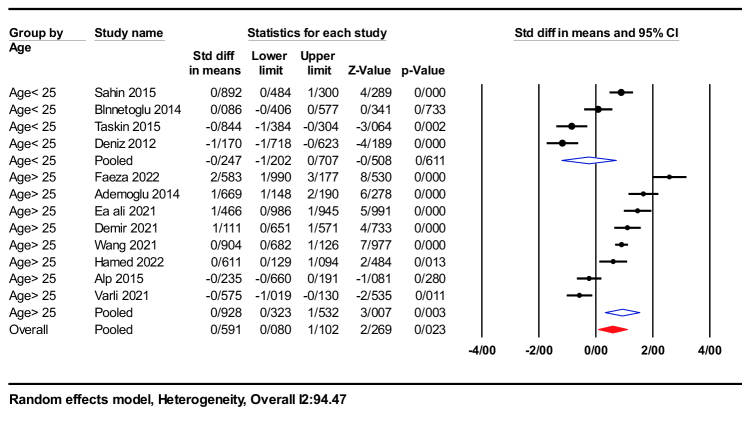
Forest plot of age ≥ 25 yr and ≤ 25 yr in subgroup analysis.

Subgroup analyses were conducted for each study based on BMI, age, and homeostatic model assessment for IR (HOMA-IR). Within the age subgroup analysis, significant differences were found between women older than 25 yr (SMD = 0.92; 95% CI: 0.32-1.53; p = 0.003) and women younger than 25 yr (SMD = -0.24; 95 % CI: -1.20-0.70; p = 0.61), as shown in figure 4.

**Figure 5:**
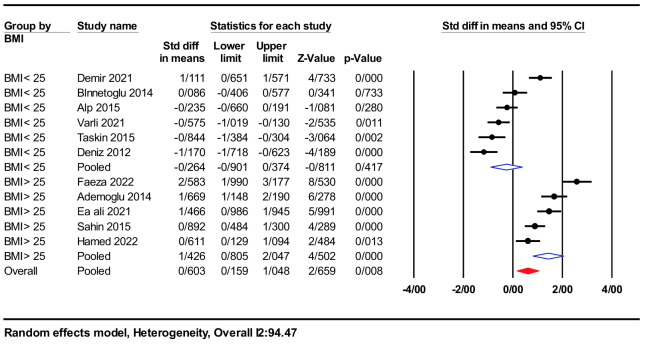
Forest plot of body mass index ≥ 25 kg/m^2^ and body mass index ≤ 25 kg/m^2^ in subgroup analysis. BMI: Body mass index.

Subgroup analysis was performed based on studies with mean BMI either ≥ 25 kg/m^2^ or ≤ 25 kg/m^2^. Women with a BMI > 25 kg/m^2^ exhibited significantly higher nesfatin-1 levels (SMD = 1.42; 95% CI: 0.80-2.04; p < 0.001) compared to women with a BMI < 25 kg/m^2^ (SMD = -0.26; 95% CI: -0.90-0.37; p = 0.41), as illustrated in figure 5.

**Figure 6:**
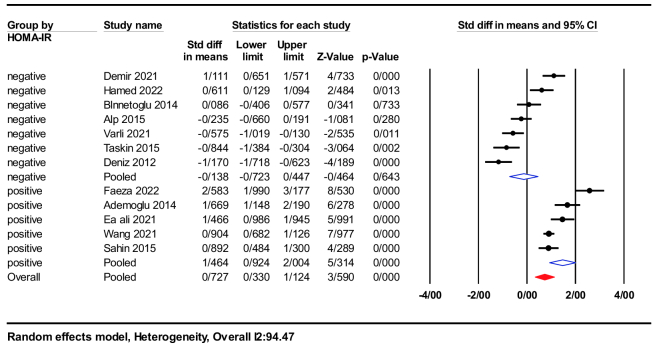
Forest plot of positive/negative HOMA-IR in subgroup analysis.

A subgroup analysis was conducted based on studies with a mean HOMA-IR of either ≥ 2.9 (positive for IR) or ≤ 2.9 (negative for IR). Participants with HOMA-IR > 2.9 (SMD = 1.46; 95% CI: 0.92-2.00; p < 0.001) had significantly higher serum nesfatin-1 levels than women with HOMA-IR < 3 (SMD = -0.13; 95% CI: -0.72-0.40; p = 0.64), as demonstrated in figure 6.

### Meta-regression analysis

**Figure 7:**
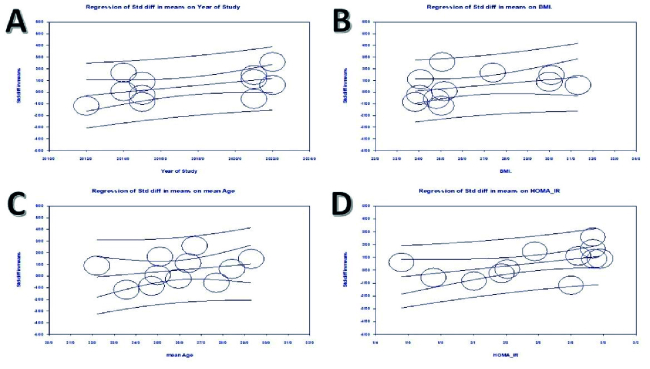
Meta-regression of circulating nesfatin-1 levels in women with PCOS Based on: A) Year of study, B) BMI, C) AGE, D) HOMA-IR.

Meta-regression analysis revealed no considerable association between nesfatin-1 levels and the year of study (meta-regression coefficient: 0.147; 95% CI: 0.01 - 0.38, p = 0.08), BMI (meta-regression coefficient: 0.154; 95% CI: -0.07 - 0.38, p = 0.17), or mean age (meta-regression coefficient: 0.153; 95% CI: -0.17 - 0.48, p = 0.35). Nevertheless, HOMA-IR was significantly correlated with TSH (meta-regression coefficient: 1.16; 95% CI: 0.09 - 2.24, p = 0.03) (Figure 7).

### Publication bias and sensitivity analysis

**Figure 8:**
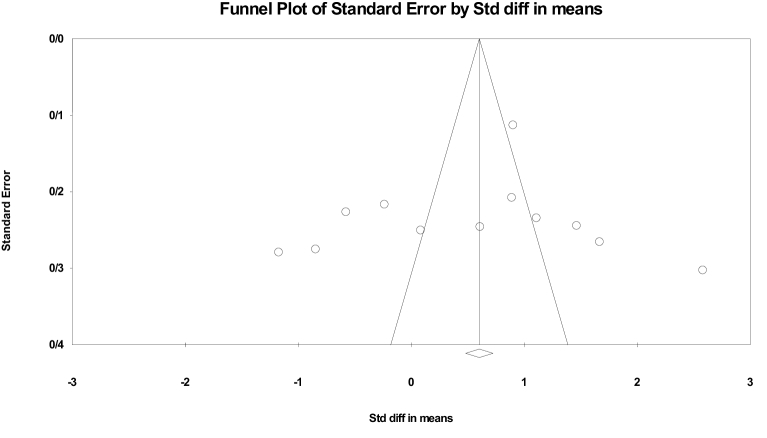
Funnel plot of standard error by standard differences in the means of serum nesfatin-1 level.

Begg's and Egger's test were utilized to evaluate publication bias in the studies included in the meta-analysis. Our findings did not demonstrate the presence of publication bias in the meta-analysis for SMD of nesfatin-1 level (Begg's test: p = 0.89; Egger's test: p = 0.61) (Figure 8). We conducted sensitivity analysis to ensure the dependability and consistency of our results, the overall effect size remained unchanged after eliminating individual studies, indicating that our analysis was dependable.

## 4. Discussions

PCOS is a prevalent reproductive condition with substantial health, psychological, and economic implications for women and society (31, 32). Furthermore, various markers have been introduced to assess this condition (33, 34). The scattered and conflicting reports regarding nesfatin-1 levels in women with PCOS have led to extensive research investigating nesfatin-1 levels (9). Several studies have indicated significantly higher nesfatin-1 levels in PCOS patients than women with no PCOS (11, 21, 28). Other studies, in contrast, have found no significant differences in nesfatin-1 levels between PCOS and non-PCOS patients (9, 15, 22, 27, 30). This meta-analysis is the first study to examine the association between nesfatin-1 levels and PCOS. Our findings and analysis revealed that nesfatin-1 levels were elevated in PCOS patients compared to individuals without PCOS. Since nesfatin-1 is derived from the adipose tissue and hypothalamus within the body (1, 2), and considering the association of PCOS with obesity (3), it is plausible that the correlation between nesfatin-1 and PCOS can be elucidated through this endocrine pathway. However, more research is necessary to comprehensively understand the cellular and endocrine mechanisms implicated in this metabolic pathway. Subgroup analysis revealed a significant increase in nesfatin-1 levels in individuals aged 25 yr or older compared with individuals younger than 25 yr. In addition, studies have consistently shown that pregnant women with PCOS and advanced maternal age have higher nesfatin-1 levels, potentially affecting maternal and fetal health. These findings suggest that the combination of PCOS and older maternal age can influence nesfatin-1 production during pregnancy, potentially affecting appetite and metabolic processes in both the mother and the child (35). The implications of these findings for maternal and fetal health should be carefully considered. Similarly, individuals with a BMI > 25 had higher nesfatin-1 levels than individuals with a BMI < 25. This finding contradicts the results of the studies by Alp et al. and Demir et al., who found no correlation between nesfatin-1 levels and BMI (15, 29). The HOMA-IR index serves as a crucial measure of IR. Individuals with HOMA-IR levels above 2.9 (positive) exhibit higher levels of nesfatin-1 compared to those with HOMA-IR levels below 2.9 (negative) (36). This finding supports the research conducted by Deniz et al., who observed elevated HOMA-IR levels in individuals with PCOS (9). However, this finding contradicts the conclusions drawn by Taskin et al. and Binnetoglu et al., who found no association between HOMA-IR and nesfatin-1 levels in PCOS patients (18, 30).

We performed subgroup analysis and employed meta-regression techniques to determine the origin of heterogeneity. When a factor demonstrates a significant correlation with heterogeneity, this allows for a more accurate assessment of effect size and supports the conclusion. In particular, a meta-regression analysis was conducted for the nesfatin-1 level using research year, BMI, age, and HOMA-IR as variables. Our findings revealed that age, BMI, and research year did not correlate with nesfatin-1 levels. Still, HOMA-IR was significantly associated with nesfatin-1 levels, and its increase considerably amplified nesfatin-1 levels, ultimately leading to the heterogeneity of the studies.

There are some limitations to the current research, including the fact that the results are confined to a specific population and geographic location. Moreover, this study highlights the importance of examining the correlation between nesfatin-1 levels and the timing of blood sampling in relation to the menstrual cycle. As a result, it was difficult to analyze this relationship due to limited availability of data and potential bias in the chosen studies. In addition, non-English articles or dissertations were not incorporated into the study. Despite these limitations, this research provides valuable insight into the relationship between nesfatin-1 levels and PCOS. Future studies may extend these findings by investigating the endocrine circuits and cellular mechanisms underlying this disorder.

## 5. Conclusion

This meta-analysis is the first attempt to investigate the potential association between nesfatin-1 levels and PCOS. Our research findings indicate that individuals with PCOS exhibit elevated levels of nesfatin-1 compared to those without PCOS. This is particularly evident among individuals with higher HOMA-IR index and IR. Despite the potential limitations of the study, these findings provide crucial insights that could assist in the development of novel treatments and management strategies for PCOS in the future.

##  Conflict of Interest

The authors declare that there is no conflict of interest.
